# Supplementary material for: A UMLS-based spell checker for natural language processing in vaccine safety
Source: BMC Med Inform Decis Mak. 2007 Feb 12;7:3. doi: 10.1186/1472-6947-7-3 (PMC1805499; doi:10.1186/1472-6947-7-3)
Supplement: Additional file 13 — RAP application directory. Sets up directory for RAP (RDF application for PHP) source code [file 1472-6947-7-3-S13.gz › rap/doc/phpdoc/__filesource/fsource_ontModel__ontModelRdfsVocabulary.php.html]

xml version="1.0" encoding="iso-8859-1"?


File Source for RdfsVocabulary.php


# Source for file RdfsVocabulary.php

Documentation is available at RdfsVocabulary.php

```
1. <?PHP
2. /**
3. * ----------------------------------------------------------------------------------
4. * Class: RdfsVocabulary
5. * ----------------------------------------------------------------------------------
6. *
7. * @package     ontModel
8. */

10. /**
11. * RDFS vocabulary items
12. *
13. * <BR><BR>History:
14. * <LI>10-05-2004                : First version of this class.</LI>
15. *
16. * @version  V0.9.1
17. * @author Daniel Westphal <mail at d-westphal dot de>
18. *
19. *
20. * @package     ontModel
21. * @access    public
22. ***/
23. class RdfsVocabulary extends OntVocabulary
24. {

26. /**
27. * Answer the resource that represents the class 'class' in this vocabulary.
28. *
29. * @return    object ResResource
30. * @access    public
31. */
32. function ONTCLASS()
33. {
34. return new ResResource(RDF_SCHEMA_URI.RDFS_CLASS);
35. }

37. /**
38. * Answer the predicate that denotes the domain of a property.
39. *
40. * @return    object ResProperty
41. * @access    public
42. */
43. function DOMAIN()
44. {
45. return new ResProperty(RDF_SCHEMA_URI.RDFS_DOMAIN);
46. }


49. /**
50. * Answer the predicate that denotes comment annotation on an ontology element.
51. *
52. * @return    object ResProperty
53. * @access    public
54. */
55. function COMMENT()
56. {
57. return new ResProperty(RDF_SCHEMA_URI.RDFS_COMMENT);
58. }

60. /**
61. * Answer the predicate that denotes isDefinedBy annotation on an ontology element
62. *
63. * @return    object ResProperty
64. * @access    public
65. */
66. function IS_DEFINED_BY()
67. {
68. return new ResProperty(RDF_SCHEMA_URI.RDFS_IS_DEFINED_BY);
69. }

71. /**
72. * Answer the predicate that denotes label annotation on an ontology element
73. *
74. * @return    object ResProperty
75. * @access    public
76. */
77. function LABEL()
78. {
79. return new ResProperty(RDF_SCHEMA_URI.RDFS_LABEL);
80. }

82. /**
83. * Answer the predicate that denotes the domain of a property.
84. *
85. * @return    object ResProperty
86. * @access    public
87. */
88. function RANGE()
89. {
90. return new ResProperty(RDF_SCHEMA_URI.RDFS_RANGE);
91. }

93. /**
94. * Answer the predicate that denotes seeAlso annotation on an ontology element
95. *
96. * @return    object ResProperty
97. * @access    public
98. */
99. function SEE_ALSO()
100. {
101. return new ResProperty(RDF_SCHEMA_URI.RDFS_SEE_ALSO);
102. }

104. /**
105. * Answer the predicate that denotes that one class is a sub-class of another.
106. *
107. * @return    object ResProperty
108. * @access    public
109. */
110. function SUB_CLASS_OF()
111. {
112. return new ResProperty(RDF_SCHEMA_URI.RDFS_SUBCLASSOF);
113. }

115. /**
116. * Answer the predicate that denotes that one property is a sub-property of another.
117. *
118. * @return    object ResProperty
119. * @access    public
120. */
121. function SUB_PROPERTY_OF()
122. {
123. return new ResProperty(RDF_SCHEMA_URI.RDFS_SUBPROPERTYOF);
124. }

126. /**
127. * Answer the string that is the namespace prefix for this vocabulary
128. *
129. * @return    string
130. * @access    public
131. */
132. function NAMESPACE()
133. {
134. return RDF_SCHEMA_URI;
135. }

137. /**
138. * Answer the predicate that denotes the rdf:type property.
139. *
140. * @return    object ResProperty
141. * @access    public
142. */
143. function TYPE()
144. {
145. return new ResProperty(RDF_NAMESPACE_URI.RDF_TYPE);
146. }
147. }
148. ?>
```

Documentation generated on Thu, 7 Jul 2005 13:43:04 +0200 by phpDocumentor 1.3.0RC3
